# Multibody kinematics optimization for motion reconstruction of the human upper extremity using potential field method

**DOI:** 10.1038/s41598-025-94394-3

**Published:** 2025-03-26

**Authors:** Iman Soodmand, Sven Herrmann, Eric Kleist, Annika Volpert, Hannes Wackerle, Peter Augat, Rainer Bader, Christoph Woernle, Maeruan Kebbach

**Affiliations:** 1 Research Laboratory for Biomechanics and Implant Technology, Department of Orthopaedics, Rostock University Medical Center, Doberaner Straße 142, 18057 Rostock, Germany; 2 Institute for Biomechanics, BG Unfallklinik Murnau, Prof. Küntscher Str. 8, 82418 Murnau, Germany; 3 Institute for Biomechanics, Paracelsus Medical University Salzburg, Strubergasse 21, 5020 Salzburg, Austria; 4 Chair of Technical Mechanics/Dynamics, Faculty of Mechanical Engineering and Marine Technologies, University of Rostock, Justus-von-Liebig-Weg 6, 18059 Rostock, Germany

**Keywords:** Inverse kinematics, Motion reconstruction, Marker tracking, Multibody kinematics optimization, Soft tissue artifact, Upper extremity, Biomedical engineering, Mechanical engineering

## Abstract

Motion reconstruction provides essential inputs for analyzing human movement through musculoskeletal simulations. To reconstruct joint angles from motion capture data, several multibody kinematic optimization methods have been developed. However, a computationally efficient method yet simple to implement while ensuring consistent kinematics at all levels is lacking. Here, we propose a potential field method generated by virtual spring-dampers connecting measured-derived skin markers to segment-fixed model points to reconstruct motion in a forward dynamic manner by solving the equations of motion. The virtual spring-damper forces move the mechanical system to minimize the elastic potential and the distance between markers during the motion. Several evaluation strategies are performed which demonstrate that the potential field method is computationally fast (2.5ms per frame) with comparable accuracy to the well-established least squares method in terms of reconstructed marker trajectories and joint angles (RMSE < 0.37 mm, 1.87°) and with low marker residuals (< 18.7 ± 12.6 mm) in line with reported ranges. Furthermore, soft tissue artifacts are compensated well compared to the simulated true values (RMSE < 1.66 mm, 3.69°). Sternoclavicular, scapulothoracic and glenohumeral rotations were reconstructed well the major trends and magnitudes of experimental measurements. We anticipate our method will pave the way for complex applications that demand reliable and rapid large-scale biomechanical analysis of human movement.

**Supplementary Information:**

The online version contains supplementary material available at 10.1038/s41598-025-94394-3.

## Introduction

Motion reconstruction from measurements is a computational approach used to calculate the human joint angles and movements required to achieve a specific motion of a body or limb^1^. Several innovative motion capturing approaches, including wearable sensors^2–4^ and marker-less motion capture systems^5–7^, have recently emerged to ease and expand the application of the motion reconstruction. However, optical motion capture remains the broadly accepted method for capturing human motion^8^. In this context, multibody kinematics optimization (MKO) has been frequently used to reconstruct and analyze the motion of the upper extremity derived from optical motion capturing^9^. The general principle of these approaches is to represent the skeletal structure of a subject by a kinematic chain consisting of a series of rigid skeletal segments connected by ideal joints^10^. The skeletal configuration of the subject is then identified by minimizing the distance between measured-derived skin marker trajectories and equivalent segment-fixed model points, which are subject to the same constraints^11,12^. As the approach was shown to effectively reduce soft tissue artifacts (STA) inherent to marker-based motion capturing^13^, recent studies on the kinematic estimation of the upper extremity focused mainly on further STA reduction and accuracy improvements^14–19^.

While these MKO approaches enhance the understanding of the human body kinematics, some aspects require improvement. Computationally efficient MKO methods, with decreased calculation time per frame, while remaining simple to implement, are required to efficiently analyze large datasets^20^, manage the complexity of biomechanical models with high degrees of freedom (DOF), and perform iterative processes for model refinement and accuracy improvement^14,19^. Joint velocities and accelerations are often derived by post-processing the acquired position data using finite differences, which can generate consistency issues that are even further aggravated by closed-loop constraints^21^. Alternatively, they can be obtained through elaborate extension of the optimization formulation by differentiating the Karush-Kulm-Tucker optimality conditions; however, this approach requires numerous partial derivatives of the objective function, leading to limitations regarding complexity and non-linearity^22^. Also, accurate conclusions on human movement kinetics need dynamically consistent kinematics with inertia properties in the musculoskeletal model and environmental constraints^23^. The kinematic analysis should consider the effect of external environmental constraints, e.g., ground reaction during gait analysis, fluid resistance for swimmers, and air resistance for cyclists^23,24^. Faber et al. showed the significance of the inconsistency between kinematics and environmental effects by calculating pelvis and low back kinematic trajectories that ensure consistency between whole-body dynamics and measured ground reactions^25^. Achieving this requires integrating inertia properties and interaction with the environment into the MKO method through forward dynamic analysis.

In this regard, the idea of connecting the skin markers to the corresponding model points using spring-damper units with determined constant values, the so-called potential field (PF) method in the present study, can be promising^26–31^. The conceptual idea of using a potential field to reconstruct motion from captured marker trajectories was employed in the case of the lower extremity^26^. The potential field method has been developed and adapted in various contexts, including its application by Kebbach et al. in 2020^27^, Lahkar et al. in 2021^30^, Wang, X. et al. in 2022^28^, and Wang, Y. et al. in 2024^29^, particularly for the motion reconstruction of the lower extremity. This method was expanded by Hermann et al., where it was applied to develop a kinematic multibody model of the upper extremity including the closed-loop structure of the shoulder complex^31^, which present unique challenges and biomechanical complexities in motion reconstruction^22^.

Therefore, the primary objective of the present study is to further develop the efficient MKO method, the PF method^31^, based on the forward dynamics that allows for considering the improvements in a single framework to analyze the upper extremity multibody model in terms of realistic reconstructed kinematics. To fully establish this method, the second objective of the present study is to assess the efficiency, reliability, and robustness of the proposed PF method for motion analysis of the human upper extremity by evaluating marker residuals, comparing the method with a well-established MKO approach based on a least squares (LS) objective function, assessing STA compensation using simulated motion, and validation against ground-truth kinematics provided by in vivo measurements^9^.

## Results

**Fig. 1:**
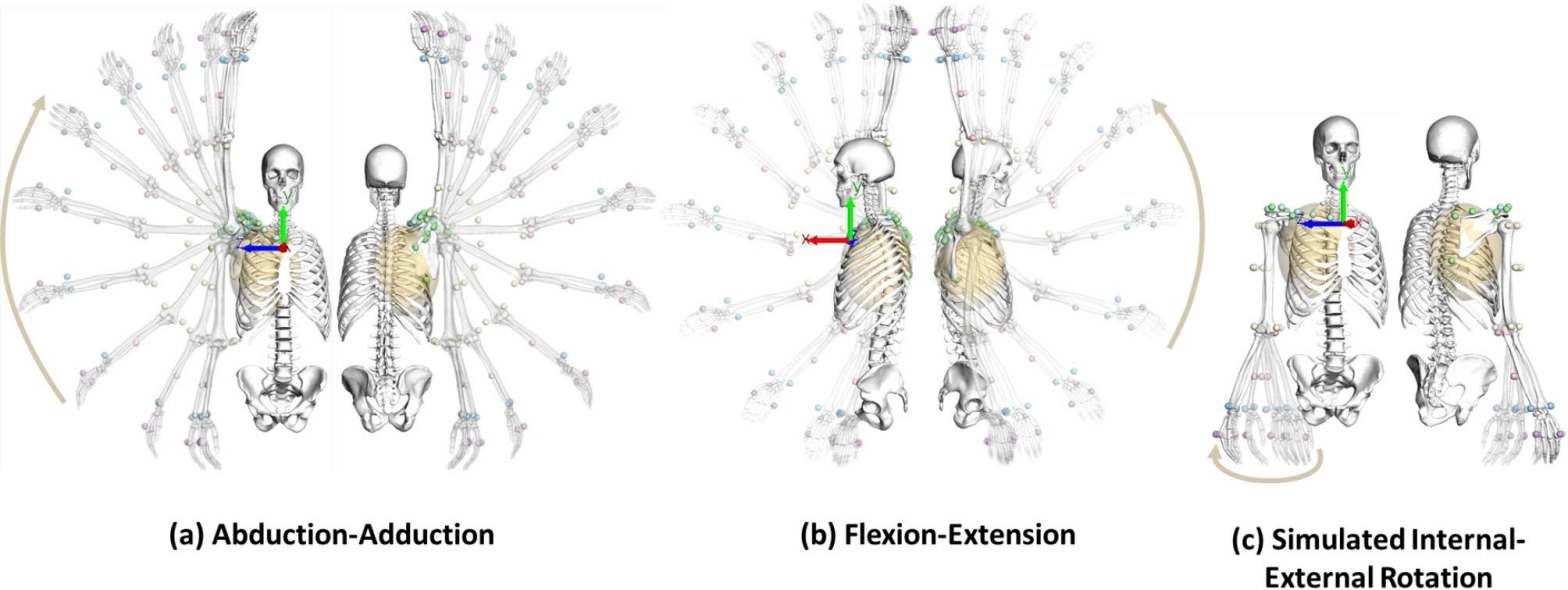
Reconstructed motions using the potential field method. Reconstructed upper extremity kinematics by the potential field method during (**a**) full arm abduction, (**b**) flexion, and (**c**) simulated internal-external rotation are shown at various time frames throughout a representative half-cycle of motion. Small spheres depict the position of skin markers at different time frames.

The proposed PF method was used to reconstruct five cycles of the captured arm abduction-adduction, five cycles of the captured arm flexion-extension, and 12.5 cycles of the simulated internal-external rotation (SIER), each simulating 25 s of motion. All bone segments and corresponding model points steadily followed the skin marker trajectories. The movement of segments and markers for a half cycle of each motion scenario is illustrated in Fig. 1 at sequential time frames. Additionally, to enhance understanding and provide a clearer visualization of the reconstructed motions, animations for a full cycle of each motion scenario are included in the supplementary material.

### Sensitivity analysis on spring-damper constants

**Fig. 2:**
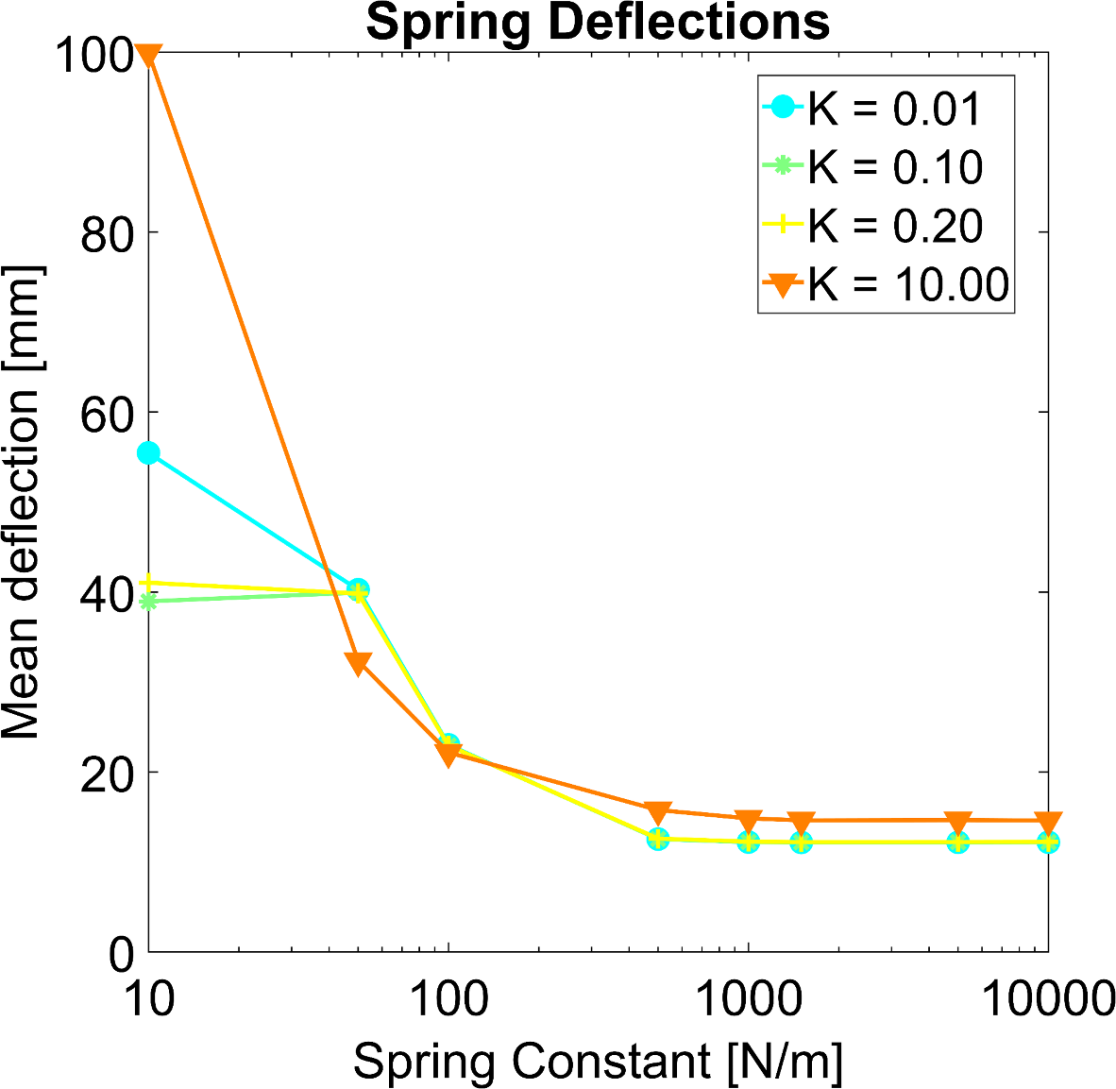
Sensitivity analysis of spring constants ci and damper-spring ratio k on mean spring deflection over all markers and all time frames. Each point shows one simulation with a different set of spring-damper constants.

Spring deflections consistently reached convergence with spring constants beyond 1000 N/m indicating the greatest achievable tracking quality (Fig. 2). The level of convergence started to rise for damper-spring ratios, $k={\text{d}}_{i}/{\text{c}}_{i},$ between 0.01 and 0.2. Increasing the k value to 10 resulted in large spring-damper forces, which exert high energy into the system, causing reconstruction inaccuracies. Therefore, uniform spring constants of $\:{\text{c}}_{i}=1500\:\text{N}{\text{m}}^{-1}$ and damping constants of $\:{\text{d}}_{i}=\:300\:\text{N}{\text{s}}{\text{m}}^{-1}$ with $\:k=\:10\:\text{s}$ were sufficient for stable and accurate subsequent analyses.

### Computational effort

The simulations for both PF and LS methods were performed on a desktop PC with the same configuration, using a single processor with a 3.30 GHz CPU and 32 GB of RAM. The wall clock time as the indicator of computational effort for the simulation of each case, as well as residual and root-mean-square errors (RMSE), are presented in Table 1.

**Table 1:** Computational effort and reconstruction errors.

Scenarios	Number of input time frames	Simulation end time (s)	Wall clock time*		Maximum Mean residuals (mm)	Maximum residuals (Marker)	Maximum RMSE (Marker)
			PF (s)	LS (s)			
Abduction-Adduction	4,541	25	18	435	18.0 ± 12.0	45.6 mm (TS1)	28.4 mm (GH)
Flexion-Extension	4,940	25	16	490	18.7 ± 12.6	49.0 mm (GH)	28.7 mm (GH)
Simulated Internal-External Rotation	96,001	30	32	5,057	4.0 ± 5.9	26.5 mm (GH)	11.8 mm (GH)

Comparison of the computational runtime required for motion reconstruction of full arm abduction-adduction, flexion-extension and simulated internal-external rotation motion scenarios using both the potential field (PF) and least squares (LS) methods. Comparison of the maximum of mean residuals over all markers as well as comparison of maximum residuals and maximum root-mean-square errors (RMSE) and the corresponding markers. GH stands for glenohumeral marker.

*It should be noted that the performance of the LS method was evaluated using an in-house developed code, which could potentially impact the fairness of the computational effort comparison between the two methods.

The calculation using the PF method was about 24, 30, and 158 times faster than the LS method for the abduction, flexion, and SIER motions, respectively (Table 1).

### Evaluation using reconstruction error

**Fig. 3:**
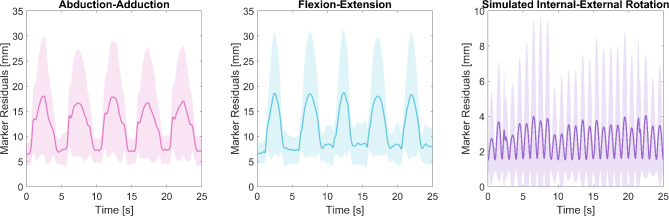
Marker residuals (mean ± standard deviation) for different motion scenarios. Residuals estimated between skin markers and model points during five subsequent motion cycles of full arm abductions, five cycles of flexions, and 12.5 cycles of simulated internal-external rotation averaged over all markers for each motion scenario.

The absolute values (norm of the x, y, and z components) of virtual spring deflections, which show the distance between the skin markers and model points, were defined as the marker residual or tracking deviation. The mean ($\:{\text{E}}_{j}$) and standard deviation of this value calculated over all markers are illustrated in Fig. 3 and its maximum value is listed in Table 1 for all motion scenarios. Maximum values of the mean residuals over markers reached 18.0 ± 12.0 mm during the abduction, 18.7 ± 12.6 mm during flexion, and 4.0 ± 5.9 mm during SIER (Table 1; Fig. 3). If the residuals were not averaged over all markers and each marker was studied individually, maximum residuals for each marker reached 45.6 mm during the abduction, 49.0 mm during flexion, and 26.5 mm during SIER. Additionally, root-mean-square error over 25 s of motion between the vector of the reconstructed position of model points and the corresponding skin markers were calculated individually for each marker, and maximum values among all markers reached 28.4 mm, 28.7 mm and 11.8 mm for the abduction, flexion, and SIER motions, respectively. The glenohumeral (GH) marker, depicted in Fig. 6, showed the maximum reconstruction errors among all markers.

### Evaluation using simulated data

**Fig. 4:**
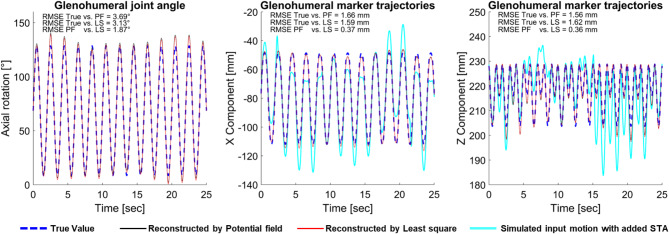
Evaluation of the potential field method based on simulated data. Comparison of the True Values of the simulated internal-external rotation (blue dashed) to the reconstructed motion by potential field (solid black) and least squares (solid red) methods, as well as to the simulated input motion of the glenohumeral (GH) marker trajectory with the added measurement noises and STA (solid cyan). The GH marker position attached to the humerus segment is shown in Fig. 6.

A simulated internal-external rotation with added noises and STA served as the input motion for the multibody model of the upper extremity. Both PF and LS methods were used to reconstruct this motion and estimate joint angles. The simulation results and the RMSE between the True Value and the calculated kinematics by PF and LS methods are shown in Fig. 4 for the axial rotation component of the glenohumeral joint angle and the RMSE were 3.69° and 3.13°, respectively. Also, the RMSE for the reconstructed glenohumeral marker displacement (the coordinate system is shown in Figs. 1 and 6) by PF and LS methods were 1.66 mm and 1.59 mm for the X component and 1.56 mm and 1.62 mm for the Z component, respectively (Fig. 4). Supplementary Fig. S1 in the supplementary materials provide a more comprehensive insight to the deviation between the True Value and reconstructed glenohumeral joint angles using PF and LS method over time.

### Validation against reference data

**Fig. 5:**
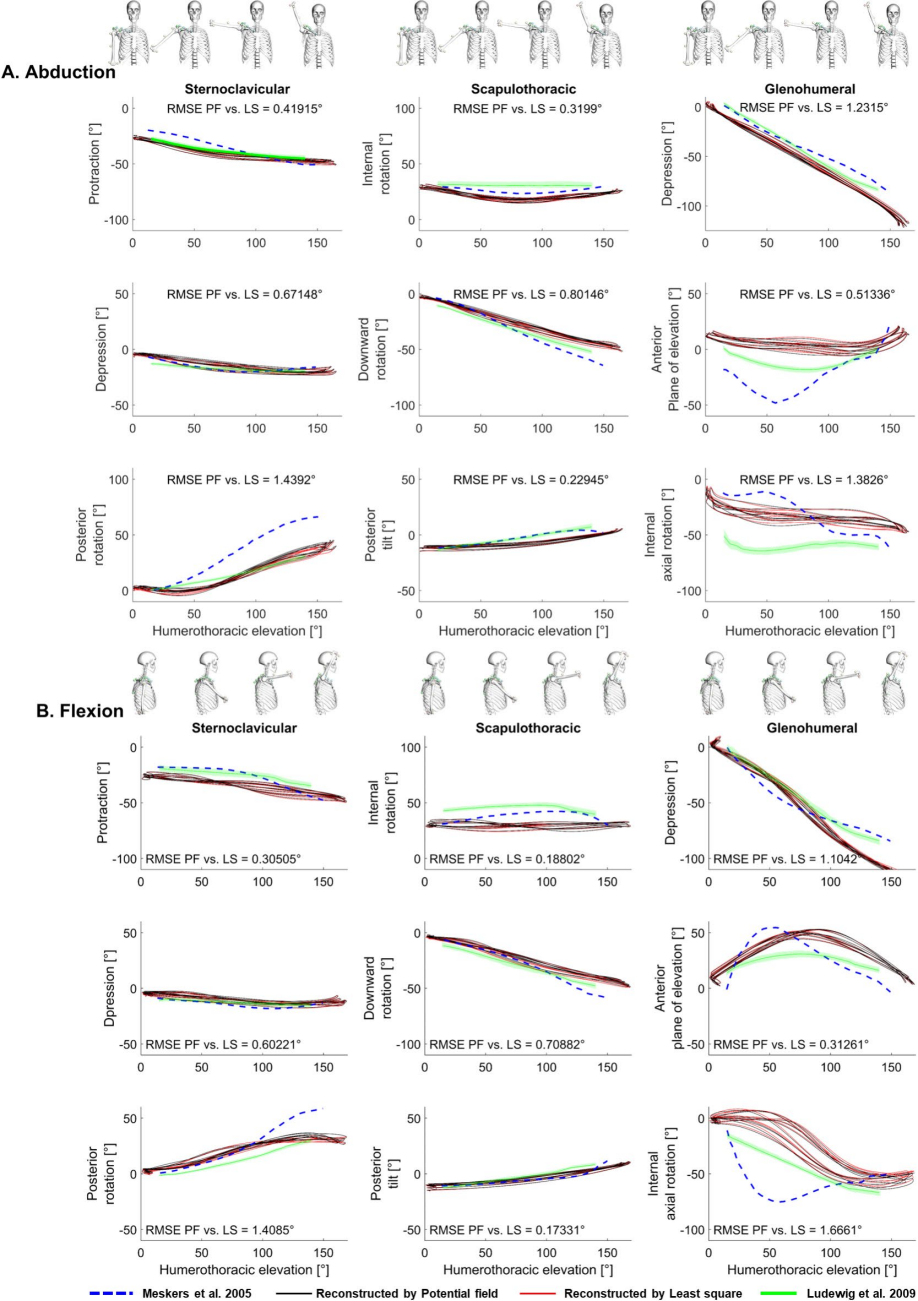
Validation of the reconstructed joint angles using the potential field method against in vivo measurements. Reconstructed sternoclavicular, scapulothoracic, and glenohumeral rotation for full arm abduction (**a**) and flexion (**b**) against humerothoracic elevation by both potential field (PF, solid black) and least squares (LS, solid red) methods in comparison to measurements by electromagnetic tracking^33^ and intracortical bone pins^32^.

The PF method was able to well reproduce major trends and magnitudes of ground-truth in vivo measurements from literature^32,33^, for sternoclavicular, scapulothoracic and glenohumeral rotations throughout arm abduction and flexion motion scenarios (Fig. 5). Also, representative reconstructed velocity and accelerations can be found in the supplementary Figs. S4 and S5. These values were close for PF and LS methods, as compared in Fig. 5, with the RMSE of less than 1.44° and 1.67° between the methods for abduction and flexion, respectively. Additionally, supplementary Figs. S2 and S3 show the time history of the deviation between the reconstructed joint angles by the PF and LS methods.

## Discussion

In the present study, a multibody kinematic model of the human upper extremity was developed to directly reconstruct the joint kinematics from measured-derived skin marker trajectories using a PF method (Fig. 1). The presented method can be interpreted as minimizing the potential energy of implemented virtual spring-damper units. In addition, several evaluation strategies were employed to assess the robustness and performance of the PF method for the reconstruction of different captured and simulated motion scenarios. In this context, we followed the systematic approach for evaluating the MKO algorithms recommended by Begon et al.^9^. The PF method showed efficiency and accuracy in its evaluation based on several criteria, including marker residuals, comparison to a well-established MKO approach (i.e., LS method), STA compensation compared to True Values, and validation against ground-truth bone kinematics. Although the present study applies the PF method to the human upper extremity, the method can also be efficiently considered for the lower extremity^27^ and the whole body.

Despite the available MKO approaches showing satisfactory reconstruction results and performance, the PF method offers several advantages. Unlike other MKO approaches, for example, the LS method, the PF method calculates the accelerations required for inverse dynamic analysis in musculoskeletal simulations directly from the captured motion trajectories by solving the equations of motion without the formulation of an additional constrained optimization problem. Since the joints and closed-loop constraints are integrated into the equations of motion, solving these equations ensures that the reconstructed motion respects them and is consistent at all kinematic levels. Supplementary Figs. S6 and S7 and supplementary animations illustrate the reconstructed motion of the closed-loop kinematic chain of the shoulder complex on the scapula during half a cycle of abduction motion.

Concerning the computational effort, the PF method is computationally fast and needs an average of 2.5 milliseconds per time frame (Table 1) to reconstruct the motion of a closed-loop upper extremity model compared to 82.6 milliseconds per time frame average for the LS method in the present study. Despite the authors trying to optimize the performance of the LS method, this meaningful difference can be attributed to the type of solver that the authors used in the present study to solve the LS optimization problem or if the written computer code is optimized in terms of efficiency. However, the optimization-based nature of the LS method requires iterative minimization of an objective function of, e.g., 13 design variables (joint angles) in the present study at each single time frame, which decreases the efficiency. Also, optimization-based methods require selecting a proper initial guess which can further decrease the efficiency. Fohanno et al.^21^ reported the mean computational time for motion reconstruction of closed-loop systems by other methods in the range of 21.4 to 201 milliseconds. Pizzolato et al.^34^ reported the overall computational time for motion reconstruction to be an average of 5.6 milliseconds per frame by solving a weighted least squares problem in OpenSim for an unconstrained gait2392 model with 32-markers marker-set and using 8 threads in parallel. Borbély and Szolgay^35^ reported the computational effort to be 145 milliseconds per frame for a simplified upper extremity model with 7 DOF^36^ in OpenSim and without closed-loop constraint on the scapula and using motion data from 20 markers. Fang et al.^37^ used the OpenSim model of the upper extremity from Holzbaur including 15 DOF and 11 markers^36^, which treats the inverse kinematic problem as a weighted least squares problem. They reported the required computational time for motion reconstruction to be an average of 90 milliseconds per frame. The readers should bear in mind that each of these studies was performed within a different computational environment for different biomechanical systems with varied DOF. For detailed information, the average reconstruction time by solving the least squares problem alongside the performance of our PF method is summarized in Table S1 in the supplementary material. The difference in computational effort becomes even more pronounced when analyzing captured motions with higher resolution, such as the simulated internal-external rotation of the present study with 96,001 time frames (Table 1). Higher efficiency enables researchers, in terms of computational effort, to combine the PF method with optimization algorithms to increase the reconstruction accuracy, e.g., by calculating an appropriate set of marker weights for each specific subject or motion scenario by solving an optimization problem^14,19^.

Virtual spring-damper units in the PF method are defined between skin markers and model points where soft tissue deformation exists. By adjusting the parameters of these virtual elements, the PF method enables considering differences in anatomic regions with varied amounts of soft tissue. An example of this is the difference between the GH marker placed on the soft tissue over the deltoid muscle and a marker placed on most caudal-medial point on the ulnar styloid, where minimal soft tissue separates the marker from the bone. Therefore, non-uniform marker weighting, previously associated with improved tracking results^14,19^, can be readily considered by adjusting the corresponding spring-damper parameters. In addition, these parameters could potentially be correlated with the biomechanical properties of real-world soft tissue underlying each marker. For example, methods such as SOD analysis^38^, as discussed by Wang et al.^29^, could be used to define soft tissue stiffness. Incorporating such correlations in future studies would help integrate the biomechanical aspects of the spring-damper parameters into our PF method, further enhancing its physical relevance.

The PF method is simple and straightforward to implement. Once the kinematic chain model of the skeletal system is built and its equations of motion are derived for inverse dynamic analysis, the implementation of the PF method requires only the addition of the corresponding set of experimental markers and model points, the connecting spring-damper force elements between them, and then parametrization of these spring-damper units. Also, the rheonomic joints were used to derive the skin markers.

Human joints have internal flexibility and musculoskeletal connections that are not fully captured by simple and idealized joint definitions such as revolute or spherical joints. A simple and idealized joint definition may not accurately replicate the complex anatomical function of a human joint^39,40^. Since the PF method reconstructs the kinematics based on a forward dynamic calculation of the equations of motion, bushing elements can be modeled as force elements and added to the equations of motion, e.g., to constrain the translational DOFs of a glenohumeral joint with six DOFs. Another advantage of the PF method is to model more complex joints rather than idealized ones simply using force elements which should be examined in future studies. By incorporating more complex joint models, the PF method can potentially reduce the effects of measurement errors, particularly STA, on the reconstructed kinematics of the musculoskeletal system and its subsequent musculoskeletal analyses.

Furthermore, the proposed PF method accounts for inertial and mass forces in kinematic estimations, which is required to reconstruct physical motions consistent with the dynamics of the body. However, the level of influence of this factor on the consistency of the reconstructed motion with the subsequent inverse dynamic simulations should be examined in future studies^23^.

Previous studies revealed that STA can amount to 87 mm concerning the actual scapula position^41^ and 48% of the effective humeral axial rotation^42^. The reported ranges correspond well to the observed tracking deviations in the present study such that the increase during abduction, flexion and SIER scenarios can mainly be attributed to STA. The difference in magnitude between the motion scenarios aligns with the notion that STA is task-specific^14^. Maximum values of the mean residuals over markers are in line with the reported values in the literature^9,41^.

As reported by Begon et al. through a systematized review study^9^ the reconstructed glenohumeral joint rotations typically showed errors ranging from 3° to 10° during arm flexion and abduction, with the largest errors occurring in internal-external rotation^15,43^. Since we have no reference data for the abduction and flexion motions from the same subject, evaluating them is not possible. However, RMSE between the reconstructed axial rotation of the glenohumeral joint by the PF method and the True Value from the simulated data in the case of SIER is estimated by 3.69°, which is in line with the error due to STA reported by Cutti et al. to be 7.0°^42^. The maximum estimated error in comparison to the True Values using the PF method in our study was 12.43° (Fig. 4). Begon et al.^15^ reported the deviation values that characterized the misorientation in humeral kinematics with respect to the true bone kinematics to be a maximum of 14° and an average of 5.9°. Also, RMSE between the reconstructed GH marker positions in the present study by the PF method and True Value is estimated by 1.66 mm and 1.56 mm for X and Z components, respectively, which aligns well with the maximum applied STA value on marker positions of 19.78 mm and 19.84 mm (Fig. 4), respectively.

A comparison of the reconstructed values by the PF method and the well-established LS method showed qualitatively similar trends, as shown in Figs. 4 and 5, with the maximum RMSE for joint angles across different DOFs of 1.44°, 1.66°, and 1.87° for the abduction, flexion and SIER, respectively. In addition, the maximum RMSE between methods for the reconstruction of marker trajectories was 0.37 mm (Fig. 4). These results suggest that the proposed PF method performs comparably to the well-established LS method. Previous studies have reported RMSE values for joint angles and range of motion within a comparable range of 1.5° to 2.0° for a similar comparison^30^. Therefore, these comparative findings may indicate the robustness and reliability of the PF method in accordance with the previous well-established and widely accepted motion reconstruction methods.

Comparison to in vivo measurements^32,33^ demonstrated overall good reproduction of realistic clavicular, scapular, and humeral motions (Fig. 5). This supports previous findings that accurate scapular kinematics can even be sufficiently represented by a segment-based model^17^. The discrepancies in the results can be mainly attributed to different cases of the present study and the literature in terms of anthropometric data, established local reference frames, marker protocols, and performed motions. Such deviations are also evident between the studies of Ludewig et al.^32^ and Meskers et al.^33^.

The present study has some limitations. It is known that STA is both subject- and task-dependent^44^. One major limitation of our computational study is that the PF method was evaluated for only three primary motion scenarios and a single subject. A study involving a larger cohort is essential for different model parameters (parameters calibration), as closed-loop constraints are sensitive to the model geometry, e.g., varying clavicle lengths among subjects^45^. Also, investigating inter-individual and -task variabilities with several subjects for daily motion scenarios and complex motion scenarios such as reaching for objects and throwing actions are important topics for using the presented method in future studies to further evaluate its practical application. The PF method was evaluated only on the position level in the present study. A meaningful comparison of skeletal velocities and accelerations would have required accurate and consistent measurements of these quantities (e.g., by using accelerometers on intracortical bone pins) which were not included in the published in vivo data^32,33,46,47^. The skeletal representation was based on a generic model with manual calibration of segmental dimensions and segment-fixed model points. A more efficient representation may be achieved by incorporating parameter identification algorithms^11,12^, which may further influence the accuracy of the motion reconstructions. Equipping motion reconstruction methods with such automated processes will enable the field of human movement analysis to minimize human modeling errors, make the pre-processing stage more efficient, ensure the consistency of each calibration, and better utilize modern, data-intensive machine-learning techniques. This approach helps the development of accurate, data-driven models to predict, prevent, and personalize treatments for movement impairments^23^. Additionally, it enhances the adaptability of the motion reconstruction to a broader range of individuals and motion scenarios. No reference kinematic as ground truth was measured, which can be captured by e.g., cortical bone pins placed in the same subject of the present study, enabling comparison of results and evaluating the ability of the PF method to compensate for STA based on real captured motion. Furthermore, the performance of the PF method can be compared to other approaches such as Kalman smoothing^48^ or multibody kinematics optimization with marker projection^15^.

Further blind validation of the PF method using datasets with both ground-truth bone and skin marker kinematics as provided by Cereatti et al.^49^, and repeatability of the results is essential^9^. The impact of other modeling choices on the estimated kinematics by the PF method, such as GH joint DOFs or scapular constraint definition, should also be studied, which may yield different results^15,17,50^. Refining the method through sensitivity analysis, e.g., on the integrator and its settings to reduce estimated RMSE values further, particularly for applications requiring higher precision such as clinical motion analysis, should also be considered as an essential next step. In addition, the advanced modeling of soft tissue behavior, as proposed by Wang et al.^29^, and the optimization of marker registration to the model^23^ represent promising directions for improving the accuracy of reconstruction. Furthermore, more realistic STA models, such as those discussed in^51,52^, should be incorporated instead of simplified models used in the present study to provide a more comprehensive assessment of the method robustness, reliability and practical applicability in realistic scenarios.

In conclusion, this study presented a potential field method to efficiently reconstruct joint kinematics of the human upper extremity from the marker trajectories for full arm abduction, flexion and internal rotation based on real motion capture and simulated motion data. The proposed method performed computationally fast, and the estimated marker residuals were in the accepted range, reported in the literature. A comparison of the reconstructed joint angles and marker trajectories obtained by the PF method to those by the well-established least squares method showed a small maximum RMSE among all studied joints and motion scenarios. Reconstruction of the simulated True Values by potential field method, showed the ability of the method to compensate the STA to an acceptable extent. The reconstructed joint angles also follow the pattern of joint angles from the ground-truth in vivo measured data. Further evaluations based on the ground-truth data and sensitivity analysis on the parameters of the method should be conducted to enhance the reliability and robustness of the methodology. Our findings demonstrate the utility of our method, establishing it as a viable tool in a wide range of applications of motion reconstruction and kinematic analysis for the whole body.

## Materials and methods

### Data acquisition and motion capturing

After creating a study protocol that documented the specifications for the subject as well as the setup and procedure of the motion analysis one male subject (27a, 177 cm, 68.4 kg) without any health restrictions in the upper extremity participated in the present study with the approval of the local ethics committee of the University of Rostock (A 2016 − 0161). Informed consent was signed by the participant. Based on existing marker protocols^53,54^, 21 infrared light-reflecting skin markers with a diameter of 9.5 mm (Prophysics AG, Kloten, Switzerland) were attached to bony anatomical landmarks identified by palpation and were distributed across the subject’s thorax, clavicle, scapula, upper and lower arm and the hand (more details regarding the placement are illustrated in supplementary Fig. S8). Two motion scenarios were captured using eight infrared Vicon cameras (Vicon MX T20-S, Vicon Motion Systems, Oxford, UK, sampling rate 200 Hz): full arm flexion-extensions and abduction-adductions at a self-selected and convenient speed. Each scenario included five motion cycles starting from the neutral position at rest up to the end of the range of motion. Three static measurements were additionally performed for model calibration at 0°, 90° and 180° arm abduction, where the position of the scapula was palpated, and the scapular skin markers were repositioned if necessary.

The measured trajectories were processed using a second-order Butterworth filter^11^ with a cut-off frequency of 2 Hz and transformed into a global reference system established according to recommendations of the International Society of Biomechanics^53^. The marker at the incisura jugularis was chosen as a spatially fixed reference point to neglect thorax movement. The obtained marker trajectories were interpolated by cubic splines for smooth differentiation, normalized in time and analytically differentiated twice. To scale and calibrate the geometry of the model segments, magnetic resonance imaging scans of the subject’s upper extremity with the attached markers were recorded and reconstructed to obtain 3D bone geometries and the position of the skin markers. Subsequently, the relative distance between the skin markers and the corresponding anatomical landmarks on bones was determined.

### Topology of the human upper extremity model

**Fig. 6:**
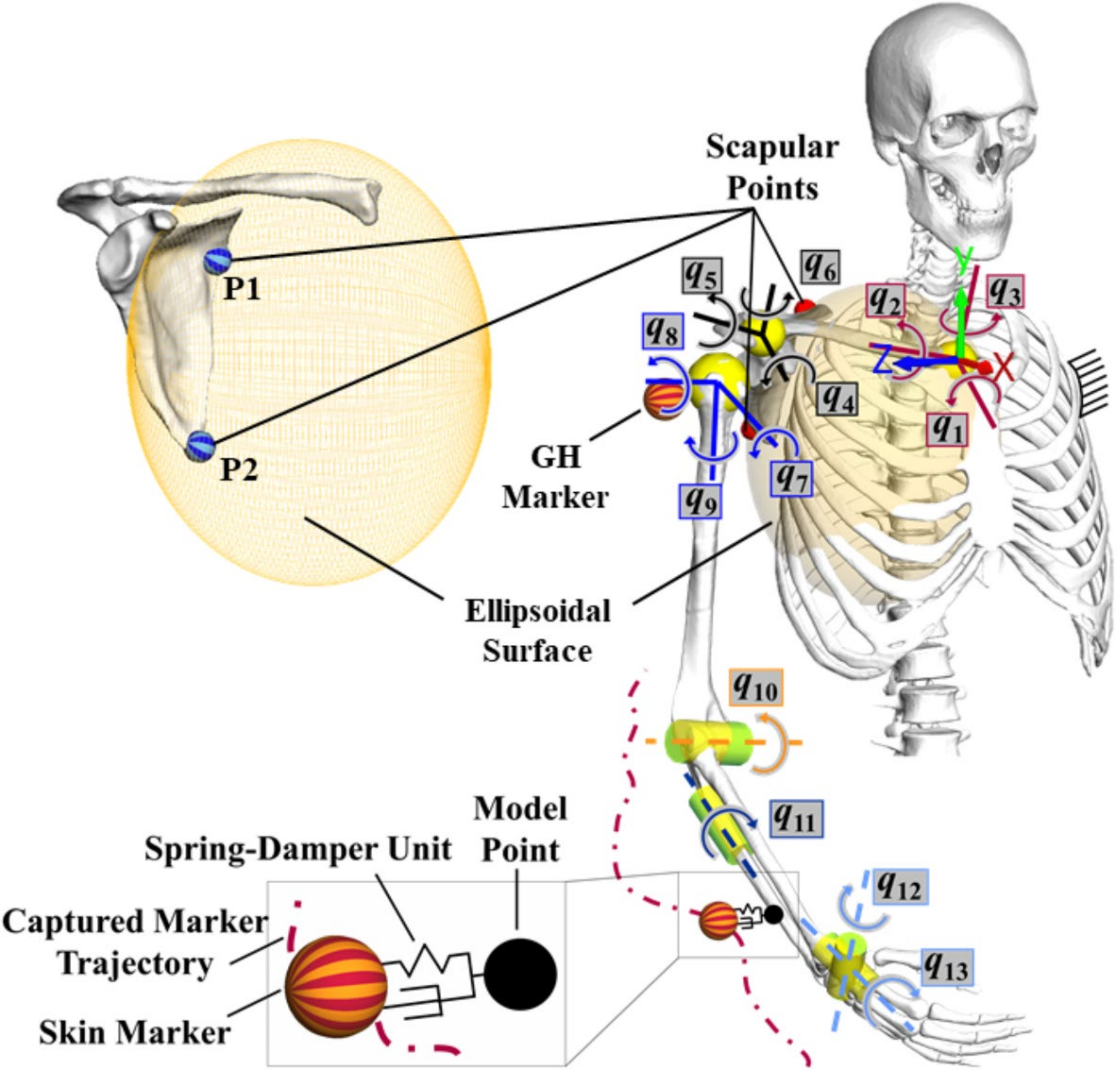
Topology of the upper extremity kinematic chain and the elements of the potential field method. It illustrates the m = 13 joint coordinates, $\:\mathbf{q}={[{q}_{1}\dots\:{q}_{13}]}^{T}$, and kinematic loop of the shoulder girdle closed by the constraints of scapula-fixed points $\:{P}_{1},{P}_{2}$ sliding on the thorax-fixed ellipsoidal surface. This multibody model is used in a forward dynamic simulation to estimate joint kinematics based on applied virtual forces generated following the deflection of the virtual spring-damper units between the skin markers and model points. The coordinate system in red (X), green (Y), and blue (Z), located on top of the manubrium of the sternum, shows the global coordinate system. Yellow spheres show spherical joints and yellow cylinders show revolute joints. GH stands for glenohumeral. It should be noted that only a limited number of representative markers are shown in this figure for model definition, and motion data from 21 markers are used to drive the model kinematics, as shown in Fig. 1.

The model topology was set up in the multibody software SIMPACK v2022x (Dassault Systèmes, Vélizy-Villacoublay, France). It consists of six movable rigid bodies (clavicle, scapula, humerus, ulna, radius and hand), and the thorax is assumed to be ground-fixed (Fig. 6). Skeletal segment geometries were derived from the male Visible Human dataset^55^ and uniformly scaled onto the 3D reconstructed bone geometries of the subject obtained from the magnetic resonance imaging scans. Local reference frames for each segment and sequence of rotations were defined according to International Society of Biomechanics recommendations^53^.

Joint rotation centers and axes were obtained by fitting spheres or cylinders into articulating surfaces. Only the glenoid rotation center was defined with respect to a glenoid-based reference system^46^ to match the humeral head dimension. The sternoclavicular and acromioclavicular joints were defined as spherical joints^50^ (Fig. 6). The glenohumeral joint was modeled by a kinematic sub-chain consisting of three revolute joints with co-intersecting axes representing a spherical joint. The articulations between the ulna and humerus were represented by a revolute joint, which enables flexion/extension of the forearm. Radius is connected to the ulna through a revolute joint representing the pro-/supination of the hand^16^. The radiocarpal joint was modeled as a cardan joint with two DOFs^50^.

The kinematic chain of the shoulder girdle was closed between the thorax and scapula utilizing two holonomic constraints (j = 1, 2), allowing the scapula to glide over the surface of the thorax^17,56,57^ (Fig. 6). Each constraint ensures that scapular points $\:{{P}}_{j}$ remain on an ellipsoidal surface approximating the thorax with coordinates given in the vector $\:{\mathbf{r}}_{{\text{e}}_{j}}$. The dimensions of the ellipsoid were functionally calibrated using the static posture measurements from the subject at different arm elevations^17,57^. The constraints were formulated in terms of $\:\mathbf{q}$ at the position, velocity and acceleration levels

1 $${g}_{j} \equiv {\mathbf{r}}_{{{\text{e}}_{j} }}^{{\text{T}}} {\mathbf{Ar}}_{{{\text{e}}_{j} }} - 1 = 0\;{\text{with}}\;{\mathbf{r}}_{{{\text{e}}_{j} }} = {\mathbf{r}}_{{{\text{P}}_{j} }} \left( {\mathbf{q}} \right) - {\mathbf{r}}_{{\text{E}}},$$

2 $$\dot{g}_{j} \equiv \mathbf{G}_{j} \dot{\mathbf{q}} = 0\;{\text{with}}\;{\mathbf{G}}_{{j}} = 2{\mathbf{r}}_{{{\text{e}}_{j} }}^{{\text{T}}} {\mathbf{AJ}}_{{{\text{P}}_{j} }},$$

3 $$\ddot{g}_{j} \equiv {\mathbf{G}}_{j} \ddot{\mathbf{q}} + \bar{\eta }_{j} = 0\;{\text{with}}\;\bar{\eta }_{j} = 2\left( {{\mathbf{\dot{q}}}^{{\text{T}}} {\mathbf{J}}_{{{\text{P}}_{j} }}^{{\text{T}}} {\mathbf{AJ}}_{{{\text{P}}_{j} }} + {\mathbf{r}}_{{{\text{e}}_{j} }}^{{\text{T}}} {\mathbf{A\dot{J}}}_{{{\text{P}}_{j} }} } \right){\mathbf{\dot{q}}},$$

where $\:\mathbf{A}\in\:{\mathbb{R}}^{\text{3,3}}$ denotes the ellipsoidal semi-axes summarized in a diagonal matrix, $\:{\mathbf{r}}_{{E}}$ ∈ $\:{\mathbb{R}}^{3}$ the global position vector of the ellipsoid, $\:{\mathbf{J}}_{{\text{P}}_{j}}\in\:{\mathbb{R}}^{3,m}$ the Jacobian matrix of point $\:{{P}}_{j}$ and the row vector $\:{\mathbf{G}}_{j}\in\:{\mathbb{R}}^{1,m}$ the constrained direction at the point $\:{\text{P}}_{j}$. Considering the closed-loop constraints, the model with *m* = 13 relative joint coordinates and two kinematic loops has *f* = 11 DOFs.

### Potential field method

The inherent motion reconstruction problem was defined by finding joint coordinates $\:\varvec{q}({t})$ for a given set of measured-derived skin marker trajectories $\:{\mathbf{r}}_{{\text{M}}_{i}}({t})$ such that the model kinematics reconstruct the recorded skeletal motion of the subject. In the present study, skeletal kinematics were reconstructed by a PF method that transfers measured-derived skin marker trajectories onto the joint kinematics in a forward dynamic manner by solving the equations of motion. More precisely, the potential field was generated by *n* = 21 virtual linear spring elements complemented by damper elements for asymptotically stable tracking behavior. Figure 6 illustrates that at one end, the massless spring-damper force elements were connected to “Skin Markers” derived by measured-derived skin marker trajectories from motion capturing, and at the other end, to the same number of body-fixed “Model Points” corresponding to each skin marker. Therefore, spring-damper force elements connect corresponding skin markers $\:{M}_{i}$ and model points $\:{R}_{i}$, where the soft tissue deformation exists. This creates a virtual potential field that drives the skeletal system to move and reconstruct the captured motion scenario. The virtual spring-damper forces move the mechanical system to minimize the elastic potential during motion, thus minimizing the residuals between skin markers and model points. These forces were then used as input to the equations of motion to calculate the joint accelerations as the outputs in a forward dynamic manner. The position of each model point $\:{\text{R}}_{i}$ was calibrated with respect to the corresponding model segment in SIMPACK based on the marker placement protocol and the static posture scenarios. Also, to drive the skin markers, a rheonomic joint was defined between each skin marker and the global coordinate system by specifying the time-dependent kinematic vectors of the skin marker trajectories.

At each time frame, the present distance $\:{\text{s}}_{i}$ and distance rate $\:{\dot{\text{s}}}_{i}$ between skin marker $\:{M}_{i}$ and model point $\:{R}_{i}$ were expressed in terms of the relative joint coordinates $\:\varvec{q}\left(\text{t}\right)$ and marker position vector $\:{\varvec{r}}_{{\text{M}}_{i}}\left(\text{t}\right)$

4 $${\text{s}}_{i} = \sqrt {{\mathbf{b}}_{i}^{{\text{T}}} {\mathbf{b}}_{i} } \;{\text{with}}\;{\mathbf{b}}_{i} = {\mathbf{r}}_{{{\text{R}}_{i} }} \left( {\mathbf{q}} \right) - {\mathbf{r}}_{{{\text{M}}_{i} }} \left( {\text{t}} \right),$$

5 $$\dot{s}_{i} = \frac{1}{{{\text{s}}_{i} }}{\mathbf{b}}_{i}^{{\text{T}}} \dot{\mathbf{b}}_{i} \;{\text{with}}\;\dot{\mathbf{b}}_{{\text{i}}} = {\mathbf{J}}_{{{\text{R}}_{i} }} {\mathbf{\dot{q}}} - {\mathbf{\dot{r}}}_{{{\text{M}}_{i} }} \left( {\text{t}} \right),$$

where matrix $\:{\mathbf{J}}_{{\text{R}}_{i}}\in\:{\mathbb{R}}^{3,{m}}$ represents the Jacobian matrix of model point $\:{{R}}_{i}$. Generalized spring-damper forces related to the joint coordinates $\:\varvec{q}$, and the generated torques were then defined as

6 $$\:{\varvec{\uptau\:}}^{\text{s}}={\mathbf{J}}^{\text{T}}\mathbf{B}\left[\mathbf{C}\mathbf{s}+\mathbf{D}\dot{\mathbf{s}}\right],$$

with the transpose of the global Jacobian $\:{\mathbf{J}}^{\text{T}}={[{\mathbf{J}}_{{\text{R}}_{1}}^{\text{T}}\dots\:\mathbf{J}}_{{\text{R}}_{{n}}}^{\text{T}}]\in\:{\mathbb{R}}^{{m},3{n}}$, the unit direction vectors of the spring-damper forces described in the matrix $\:\mathbf{B}\in\:{\mathbb{R}}^{3{n},{n}}$, and spring deflections and deflection rates summarized in vectors $$\:\mathbf{s}={\left[\begin{array}{ccc}{\text{s}}_{1}&\:\dots\:&\:{\text{s}}_{{n}}\end{array}\right]}^{\text{T}}\in\:{\mathbb{R}}^{{n}}$$ and $$\:\dot{\mathbf{s}}={\left[\begin{array}{ccc}\dot{{\text{s}}_{1}}&\:\dots\:&\:\dot{{\text{s}}_{{n}}}\end{array}\right]}^{\text{T}}\in\:{\mathbb{R}}^{{n}}$$, respectively. The spring and damping constants are provided in diagonal matrices $\:\mathbf{C}=\text{d}\text{i}\text{a}\text{g}({\text{c}}_{1}\dots\:{\text{c}}_{n})\in\:{\mathbb{R}}^{{n},{n}}$ and $\:\mathbf{D}=\:\text{d}\text{i}\text{a}\text{g}\left({\text{d}}_{1}\dots\:{\text{d}}_{\text{n}}\right)\in\:{\mathbb{R}}^{{n},{n}}$, respectively. The parameters of the virtual spring-damper system were determined based on a sensitivity analysis aimed at converged marker residuals and the tracking capability of the model. This ensured that the selected values effectively constrained the model points to the measured-derived skin markers. For this, we reconstructed the motion through 32 simulations representing eight set of spring constants and four values for damper-spring ratio, in which spring constants ci were altered between 10 and 10000 N/m with damper-spring ratio k = d*i*/c*i* varying from 0.01 to 10 s (Fig. 2). The mean of spring deflections over all markers and all time frames served as output parameter.

Finally, the equations of motion were given as a set of differential-algebraic equations with the *m* joint coordinates $\:\mathbf{q}$, and two reaction force coordinates $\:\varvec{\uplambda\:}$

7 $$\:\left[\begin{array}{cc}\mathbf{M}&\:-{\mathbf{G}}^{\text{T}}\\\:-\mathbf{G}&\:0\end{array}\right]\left[\begin{array}{c}\ddot{\mathbf{q}}\\\:\lambda\:\end{array}\right]=\left[\begin{array}{c}{\varvec{\uptau\:}}^{\text{s}}+{\varvec{\uptau\:}}^{\text{c}}\\\:\stackrel{-}{\varvec{\upeta\:}}\end{array}\right],$$

where $\:\mathbf{M}\in\:{\mathbb{R}}^{{m},{m}}\:$denotes the mass matrix consisting of both bone and soft tissue mass calculated based on the regression equations from Winter^58^ and further explained in supplementary material and Fig. S9, $$\:\mathbf{G}={\left[\begin{array}{cc}{\mathbf{G}}_{1}&\:{\mathbf{G}}_{2}\end{array}\right]}^{\text{T}}\in\:{\mathbb{R}}^{2,{m}}$$ the matrix of the two constraints (see Eq. (2)) and $\:{\varvec{\uptau\:}}^{\text{c}}\in\:{\mathbb{R}}^{{m}}$ the generalized centrifugal and Coriolis forces.

In each simulation, the initial joint kinematics was calculated by applying static equilibrium in accordance with the initial neutral position of the right upper extremity of the subject. This was done prior to any calculations by adjusting the initial lengths of the springs between skin markers and model points, to have unstrained springs at the time frame zero, equations of motion, Eq. (7), were then resolved using a SIMPACK optimized DASRT integrator^59^ with root function handling, SODASRT 2 integrator, to obtain joint accelerations $\:\ddot{\mathbf{q}}\left(\text{t}\right)$ and their time integrations $\:\dot{\mathbf{q}}\left(\text{t}\right)$ and $\:\mathbf{q}\left(\text{t}\right)$. Intersegmental joints and constraints are typically incorporated into the differential-algebraic equations of motion. Solving these equations inherently considers the constraints, ensuring the resulting motion respects them and generates consistent kinematics at all levels when solving the equations of motion^60^.

### Evaluation and validation of the model-derived kinematics

There are several methods to validate and evaluate the performance of a multibody kinematics optimization method^9^. In the present study, four strategies were employed to evaluate the PF method for abduction-adduction, flexion-extension, and internal-external rotation scenarios.

#### Evaluation using reconstruction error

To assess the reconstruction quality, for abduction-adduction, flexion-extension, and simulated internal-external rotations, the mean residual error $\:{\mathbf{E}}_{j}$ between skin markers $\:{\text{M}}_{i}$ and segment-fixed model points $\:{\text{R}}_{i}$ were evaluated^11^ throughout the complete motions, defined as an average over all markers at each time frame *j*

8 $$\:{\mathbf{E}}_{j}=\sum\:_{i=1}^{n}\frac{{R}_{ij}}{n},$$

where $\:{\text{R}}_{ij}$ represents the residual error between skin marker* i* and the corresponding model point at time frame *j*, and *n* = 21 is the number of markers.

#### Evaluation using simulated data

A simulated internal-external rotation was generated according to Roux et al.^13^. For this, a sinusoidal function was used to create an internal-external rotation of the glenohumeral joint in the upper extremity model through forward kinematics by applying angular glenohumeral joint kinematics on the identical upper extremity model. This rotational driver served as the reference glenohumeral joint kinematics or the so-called “True Value”. The trajectory of the model points was then measured from this forward kinematic simulation. To replicate real measurement conditions, random measurement and STA noises adapted from Roux et al.^13^ were added to the marker trajectories of this reference kinematic to create the simulated motion. The resulting noisy marker trajectories, defined as simulated marker coordinates, were used as input to the PF method to reconstruct the joint kinematics and evaluate the performance of the method. Hereafter this is referred to as “Simulated input motion with added STA”. Further details are provided in the supplementary material and Fig. S10.

#### Comparison to Least Squares optimization

Another evaluation strategy was to compare the reconstructed kinematic results from the PF method with those obtained from an MKO approach with the least squares objective function, hereafter referred to as the LS method. The LS method stands as a robust, widely accepted MKO method that many researchers routinely employ to reconstruct joint kinematics from the measured-derived skin marker trajectories^10,22,61,62^, making it a suitable benchmark for comparison with innovative methodologies. In the present study, the LS method was applied to the identical upper extremity model for the abduction-adduction, flexion-extension, and simulated internal-external rotation scenarios to reconstruct the joint kinematics. For this, the following LS objective function was used

9 $$obj~ = ~\mathop \sum \limits_{{i = 1}}^{n} \left\| {{\mathbf{r}}_{{{\text{R}}_{i} }} \left( {\mathbf{q}} \right) - {\mathbf{r}}_{{{\text{M}}_{i} }} \left( {{t}} \right)} \right\|^{2},$$

where $\:{\mathbf{r}}_{{\text{M}}_{i}}\left({t}\right)$​ represents the vector of the measured marker positions and $\:{\mathbf{r}}_{{\text{R}}_{i}}\left(\mathbf{q}\right)$ denotes the model point positions calculated from the current joint angles $\:\mathbf{q}$. This optimization problem was implemented in Matlab R2023a (MathWorks, Natick, MA, USA) and resolved using the ‘fmincon’ optimization solver, which finds the minimum of a constrained nonlinear multivariable function^63^, subject to the ellipsoidal constraints specified in Eq. (1) to (3).

#### Validation against reference data

In vivo measurements, including intracortical pins, dynamic stereo-radiography, and biplanar X-rays, which are considered the gold standard in motion reconstruction studies, can provide reference data to assess the accuracy of results derived by numerical simulations^9^. In the present study, the reconstructed sternoclavicular, scapulothoracic and glenohumeral joint kinematics by the PF method were validated against ground-truth bone kinematics obtained using the in vivo measurements from Ludewig et al.^32^ based on the intracortical bone pins and by Meskers et al.^33^ based on the electromagnetic tracking. The performance of the PF and LS methods was compared for this reconstruction. All rotations were evaluated according to InternationalSociety of Biomechanics rotation sequences^53^, except for glenohumeral rotations using XZY Cardan sequences^64^.

## Electronic supplementary material

Below is the link to the electronic supplementary material.

Supplementary Material 1

Supplementary Material 2

Supplementary Material 3

Supplementary Material 4

Supplementary Material 5

Supplementary Material 6

Supplementary Material 7

Supplementary Material 8

## Data Availability

The original contributions presented in the study are included in the article/Supplementary Material; further inquiries can be directed to the corresponding author.

## Abbreviations

STA: Soft tissue artifacts
DOF: Degree of freedom
MKO: Multibody kinematics optimization
PF: Potential field
LS: Least squares
RMSE: Root-mean-square error
GH: Glenohumeral
SIER: Simulated internal-external rotation
