# Supplementary material for: Multibody kinematics optimization for motion reconstruction of the human upper extremity using potential field method
Source: Sci Rep. 2025 Mar 26;15:10411. doi: 10.1038/s41598-025-94394-3 (PMC11947205; doi:10.1038/s41598-025-94394-3)
Supplement: Supplementary file 8 — Supplementary Material 8 [file 41598_2025_94394_MOESM8_ESM.docx]

**1S. Results**


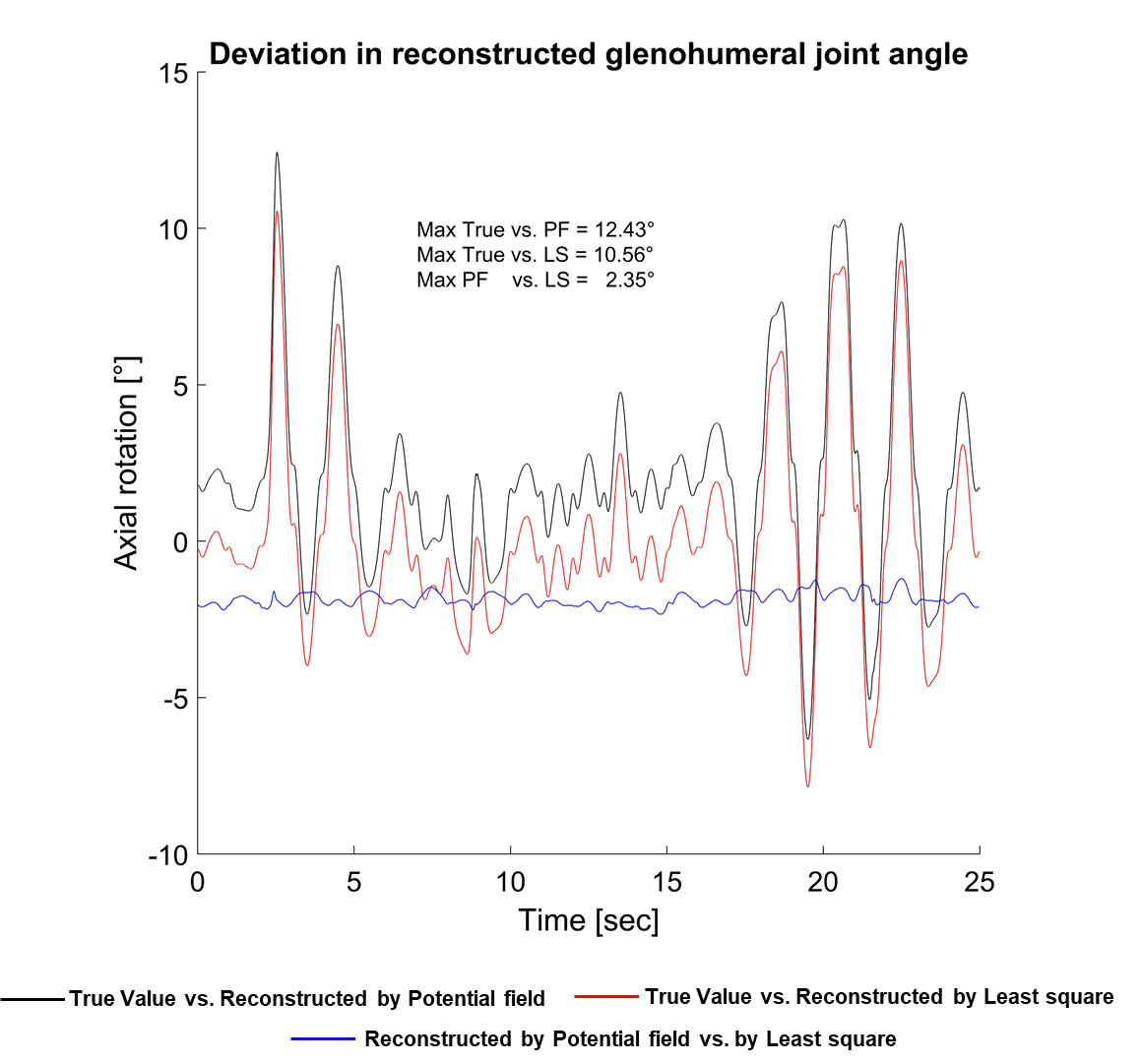


**Figure. S1 |** Time history of the deviation between the True Value and reconstructed glenohumeral joint angles using the potential field and least squares methods during the simulated internal external rotation.





**Figure. S2 |** Time history of the deviation between the reconstructed glenohumeral joint angles using the potential field and least squares methods during the abduction motion.





**Figure. S3 |** Time history of the deviation between the reconstructed glenohumeral joint angles using the potential field and least squares methods during the flexion motion.





**Figure. S4 |** Angular kinematics of the glenohumeral joint at the position (a), velocity (b), and acceleration (c) levels during five cycles of abduction-adduction motion.







**Figure. S5 |** Detailed view of the kinematics of the glenohumeral joint at the velocity (a) and acceleration (b) levels during one abduction-adduction motion cycle.


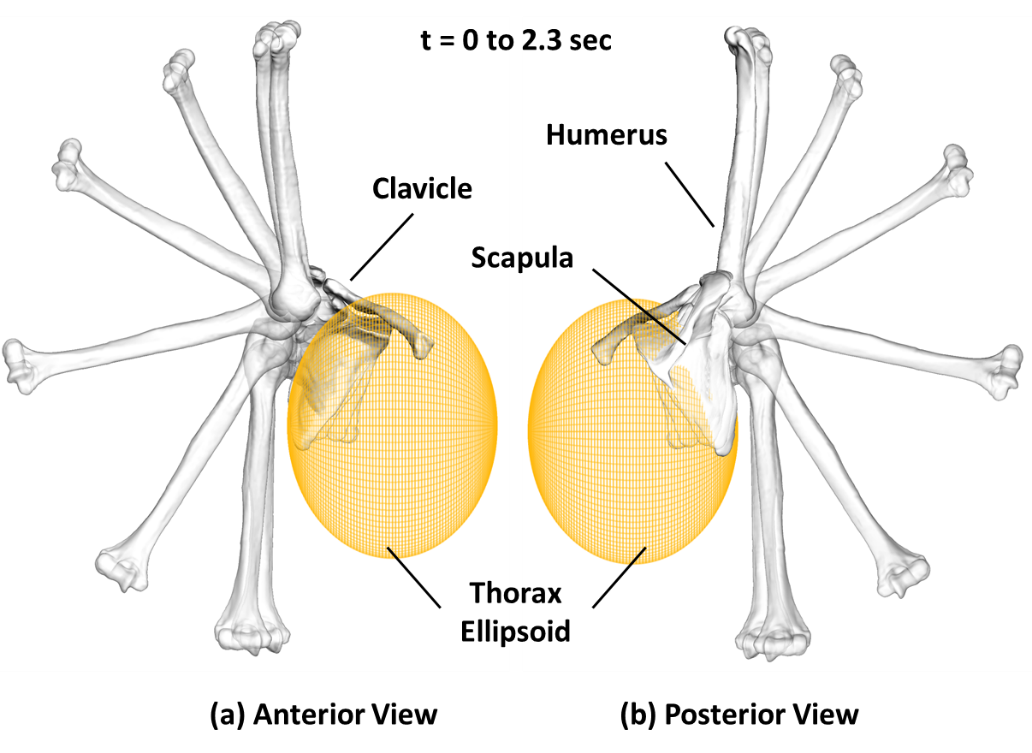


**Figure. S6 |** Overlay view of the scapula motion relative to the thorax, the ellipsoid of the thorax, and the humerus bone from anterior (a) and posterior (b) views at the same time instances from t = 0 to t = 2.3 sec, demonstrating the closed-loop kinematic chain of the shoulder complex during abduction motion.


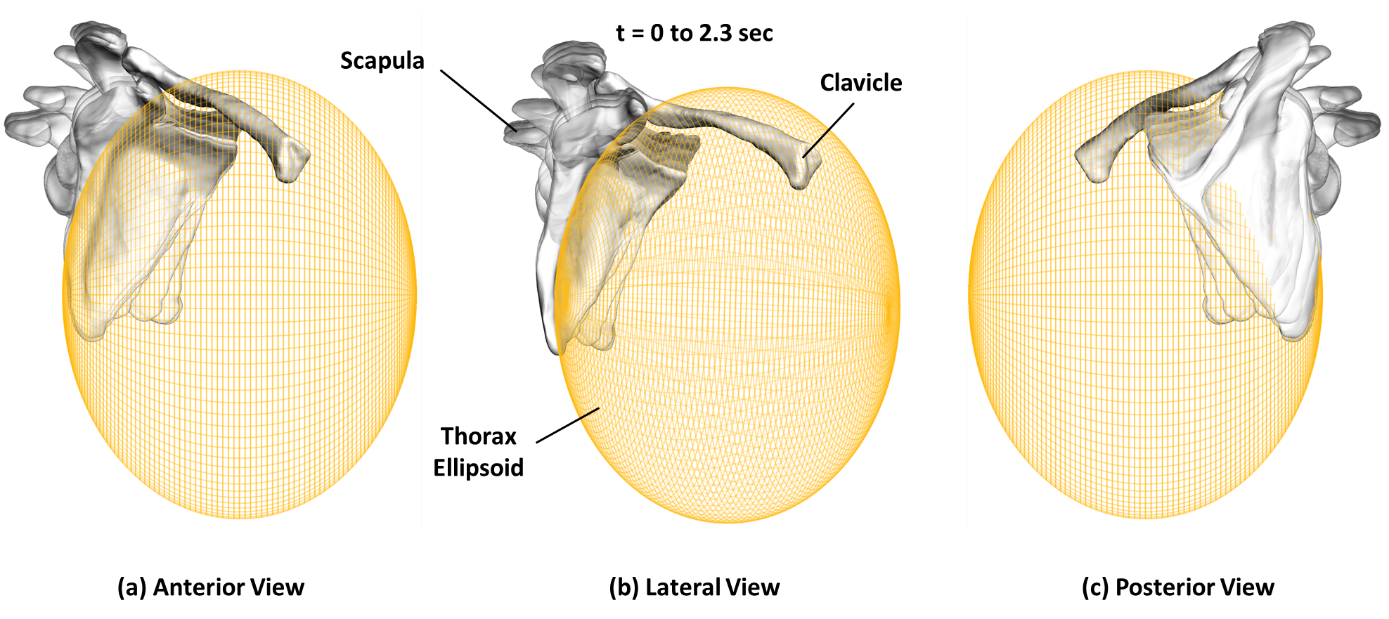


**Figure. S7 |** Detailed view of the scapula trajectory over time on the ellipsoid of the thorax from anterior (a), lateral (b), and posterior (c) views at the same time instances from t = 0 to t = 2.3 sec during abduction motion.

**Table S1 | Computational effort of the potential field and least square methods for comparing the finding of the present study with the literature**

| **Method – Study –  Extremity** | **Number of  DOF** | **Number of markers** | **Number of threads** | **PC  specification** | **Software** | **Reported computational effort (ms)** |
| --- | --- | --- | --- | --- | --- | --- |
| PF – present study - Upper extremity | 11 | 21 | 1 | 3.30 GHz CPU -  32 GB RAM | Simpack v.2022x | 2.5 |
| LS – present study Upper extremity | 11 | 21 | 1 | 3.30 GHz CPU -  32 GB RAM | Matlab v.2023a | 82.6 |
| LS – Anybody Lower extremity | 21 | 26 | 1 | 3.30 GHz CPU -  32 GB RAM | AnyBody modeling System v.7.4 | 46.3 |
| LS – Pizzolato et al. 2016 [1] – lower extremity | 23 | 32 | 8 | 2.80 GHz CPU –  8 GB RAM | Original Opensim v.3.3 | 5.6 |
| LS – Borbély and Szolgay 2017 [2] – Upper extremity | 7 | 20 | NA Multi | 2.30 GHz CPU -  64 GB RAM | Original Opensim v.3.3 | 145 |
| LS – Fang et al. 2018 [3] – Upper extremity | 11 | 15 | 1 | 2.10 GHz CPU –  8 GB RAM | Original Opensim v. Unknwon | 90 |

**2S. Material and Methods**

**2S. 1 Details of skin markers**

In the present study, the motion trajectories of 21 reflective skin markers attached to the upper extremity of the subject were captured. Our model includes 21 bone-fixed model points that correspond to each of these skin markers. Consequently, motion data from all 21 markers are used to drive the skeletal system based on the potential field method. Fig. S8 illustrates the number of markers and their positions.


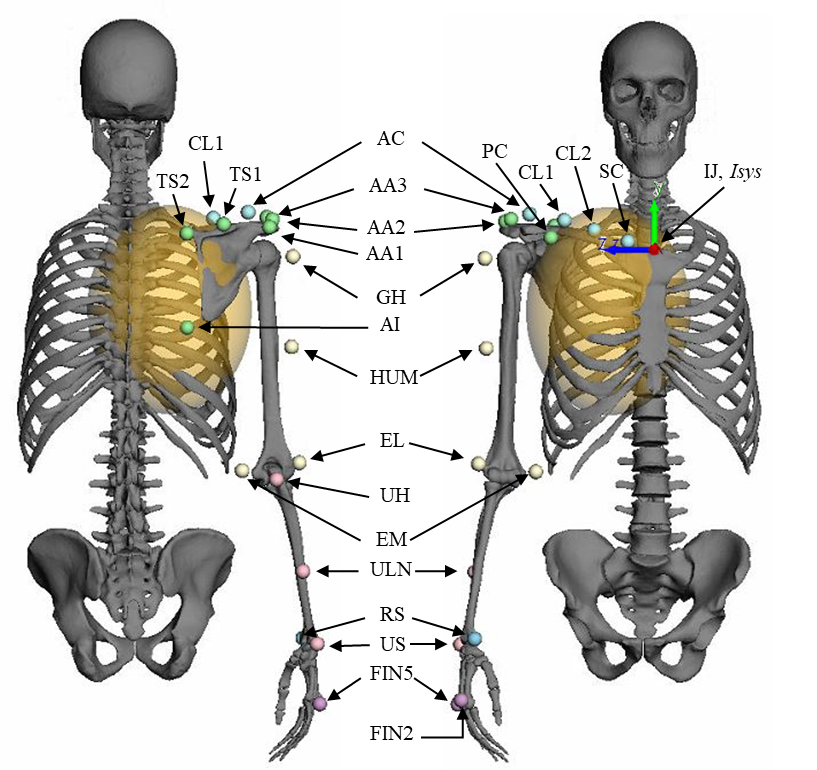


**Figure. S8 |** Position of the 21 skin markers according to the experimental marker protocol, implemented on the multi-body model of the upper extremity skeletal system**.**

**2S. 2 Calculation of mass and moment of inertia**

The PF method in the present study accounts for the inertia and mass forces when solving the equations of motion. The markers themselves are massless. However, in the PF method, the inertial properties of the anatomical structures were incorporated directly into the model segments, rather than being linked to the markers. The mass matrix was calculated based on the regression equations proposed by Winter (2009) [4]. Winter expressed the overall mass of each segment as a percentage of the total body mass. However, we did not stop here and went further by calculating the mass and inertial properties of bones and surrounding soft tissues separately to enhance the accuracy of our estimations.

For the bones, we utilized the volume of the 3D geometries and a density based on literature, as reported by Taylor et al. (2002) [5], to calculate the mass. The surrounding soft tissue, however, was modeled using simplified geometric abstracts. More precisely, the soft tissue surrounding the upper arm (humerus) and forearm (ulna and radius) were approximated as cylinders [6] as shown in Figure S9, with a density based on literature, as reported by Samuel R. Ward et al., 2005 [7]. The soft tissue of the hand segment was approximated based on a more realistic individual 3D reconstruction, as shown in Figure S9. The inertia properties were then calculated based on mathematics. Mass and moment of inertia of the soft tissue surrounding the clavicle and scapula were neglected since their contribution to the overall dynamics was determined to be minimal according to the biomechanical context of the shoulder complex.


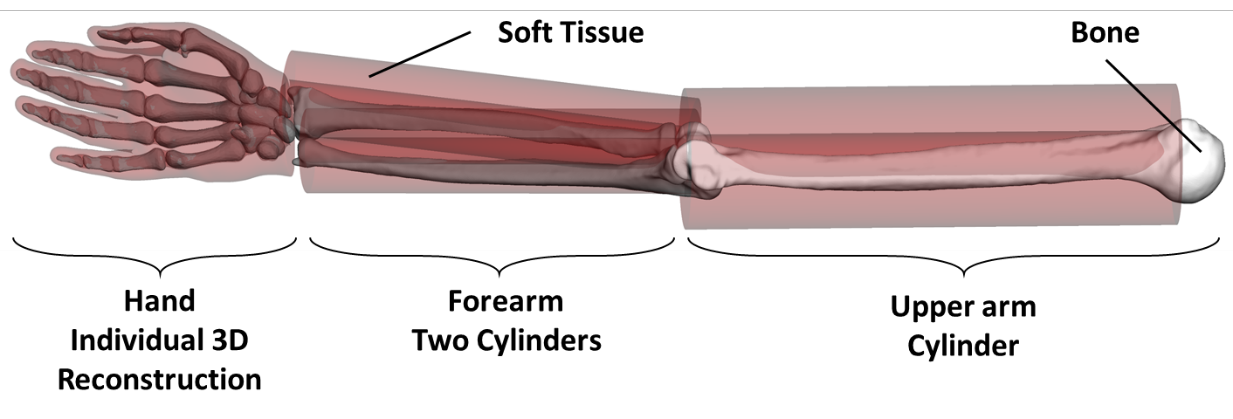


**Figure. S9 |** The moments of inertia of the surrounding soft tissue were calculated based on geometric abstracts for the upper- and forearm and individual 3d reconstruction of the hand.

**2S. 3 Generating the simulated internal-external motion**

For evaluation of the reliability of the potential field in the present study, simulated internal-external rotation was synthetically generated by computer programming and adding noises to reference noiseless marker trajectories. The framework for generating this simulated motion is visualized in Fig S10.


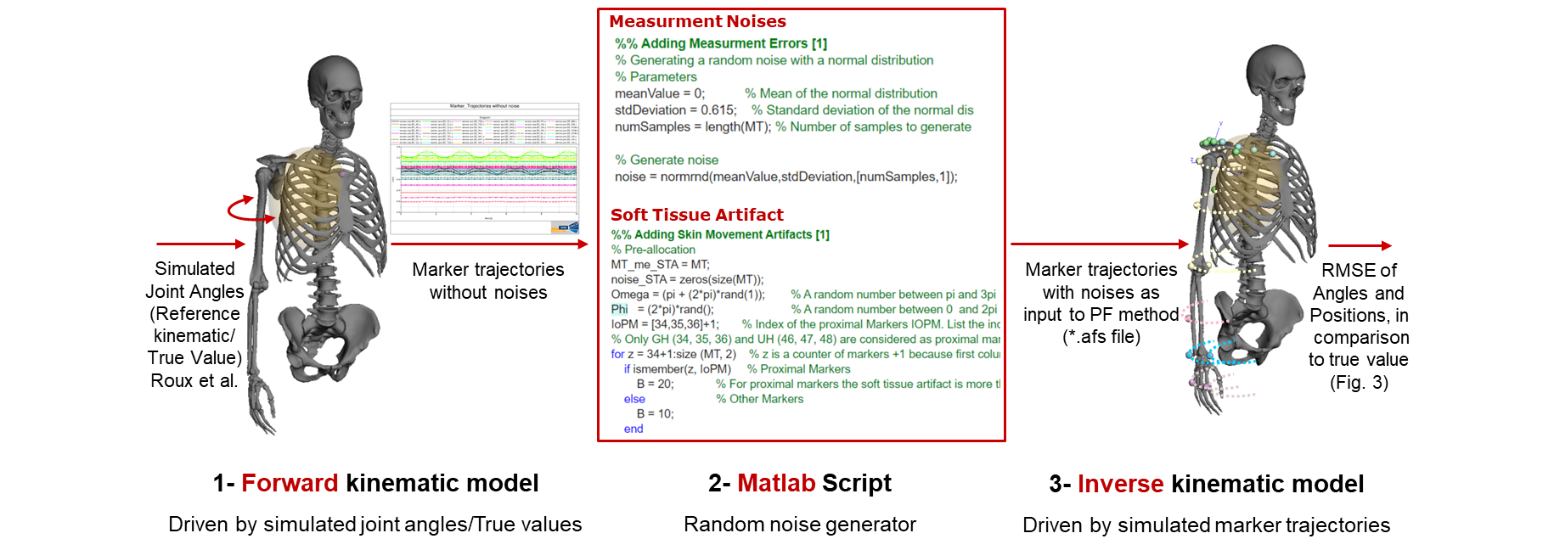


**Figure. S10 |** Framework for synthetically generating the simulated internal-external motion for evaluating the potential field method.

More precisely, a reference kinematic as virtual ground truth or the so-called “True Value”, was affected by noises that drive the joints.

$$Rot= {Rot}_{Arminit}+\pi/3\sin(2\pi0.5t )$$

with ${Rot}_{Arminit}$ the initial rotation angle, i.e. the angular configuration during the static trial. One period of the sinusoidal wave had a 50Hz sample frequency and movement duration was 25 seconds.

Two levels of noise were added to this True Value:

1. A random noise representing measurement or instrument errors with a normal distribution (mean=0mm; standard deviation=0.615mm).
2. Skin movement artifacts were simulated for marker m by a continuous noise model of the form $Asin(\omega t+\varphi)$ with $A_{m}= B_{m}\left| \sin(2\pi0.5t ) \right|$, $B_{m}\in\left\{ 10,20 \right\}$, $0.05\pi<\omega_{m}<0.15\pi$, and $\pi<\varphi_{m}<3\pi$with $A_{m}$, $\omega_{m}$, and $\varphi_{m}$ being the amplitude, frequency, and phase angle of the noise, respectively.

This led to a maximum applied virtual soft tissue value on marker positions of 19.84 mm as shown in figures (Fig. 4).

**List of supplementary animations**

1. Supplementary Animation S1_Abduction.mp4

**Animation S1 |** Reconstructed abduction-adduction motion of upper extremity by potential field method shown in anterior and posterior view at various time frames throughout a representative full cycle of motion. Small spheres depict the position of measured-derived skin markers.

1. Supplementary Animation S2_Flexion.mp4

**Animation S2 |** Reconstructed flexion-extension motion of upper extremity by potential field method shown in the right and left lateral view at various time frames throughout a representative full cycle of motion. Small spheres depict the position of measured-derived skin markers.

1. Supplementary Animation S3_Simulated internal-external rotation

**Animation S3 |** Reconstructed simulated internal-external rotation of upper extremity by potential field method shown in the anterior and posterior isometric view at various time frames throughout a representative full cycle of motion. Small spheres depict the position of measured-derived skin markers.

1. Supplementary Animation S4_Scapula movement_Anterior_View_Rev.1.mp4

**Animation S4 |** Detailed view of the scapula motion relative to the ellipsoid of the thorax from anterior view between t = 0 and t = 5 sec, demonstrating the closed-loop kinematic chain of the shoulder complex during abduction motion.

1. Supplementary Animation S5_Scapula movement_Lateral_View_Rev.1.mp4

**Animation S5 |** Detailed view of the scapula motion relative to the ellipsoid of the thorax from lateral view between t = 0 and t = 5 sec, demonstrating the closed-loop kinematic chain of the shoulder complex during abduction motion.

1. Supplementary Animation S6_Scapula movement_Posterior_View_Rev.1.mp4

**Animation S7 |** Detailed view of the scapula motion relative to the ellipsoid of the thorax from posterior view between t = 0 and t = 5 sec, demonstrating the closed-loop kinematic chain of the shoulder complex during abduction motion.

1. Supplementary Animation S7_Scapula movement_Lateral_View_2_Rev.1.mp4

**Animation S8 |** Detailed view of the scapula motion relative to the thorax, the ellipsoid of the thorax, and the humerus bone from lateral view between t = 0 and t = 5 sec, demonstrating the closed-loop kinematic chain of the shoulder complex during abduction motion.

**References**

1. Pizzolato, C., Reggiani, M., Modenese, L. & Lloyd, D. G. Real-time inverse kinematics and inverse dynamics for lower limb applications using OpenSim. Computer methods in biomechanics and biomedical engineering 20, 436–445 (2017).

2. Borbély, B. J. & Szolgay, P. Real-time inverse kinematics for the upper limb: a model-based algorithm using segment orientations. Biomedical engineering online 16, 21 (2017).

3. Fang, C., Ajoudani, A., Bicchi, A. & Tsagarakis, N. G. A Real-Time Identification and Tracking Method for the Musculoskeletal Model of Human Arm, 3472–3479.

4. Winter, D. A. Biomechanics and motor control of human movement. 4th ed. (Wiley, 2009).

5. Taylor, W. R. et al. Determination of orthotropic bone elastic constants using FEA and modal analysis. Journal of biomechanics 35, 767–773 (2002).

6. Hu, T., Kühn, J. & Haddadin, S. Forward and inverse dynamics modeling of human shoulder-arm musculoskeletal system with scapulothoracic constraint. Computer methods in biomechanics and biomedical engineering 23, 785–803 (2020).

7. Ward, S. R. & Lieber, R. L. Density and hydration of fresh and fixed human skeletal muscle. Journal of biomechanics 38, 2317–2320 (2005).
